# An Enhanced Spatial Smoothing Technique of Coherent DOA Estimation with Moving Coprime Array

**DOI:** 10.3390/s23198048

**Published:** 2023-09-23

**Authors:** Meng Yang, Yu Zhang, Yuxin Sun, Xiaofei Zhang

**Affiliations:** College of Electronic Information Engineering, Nanjing University of Aeronautics and Astronautics, Nanjing 211106, China; yangneng@nuaa.edu.cn (M.Y.); sunyuxin@nuaa.edu.cn (Y.S.); zhangxiaofei@nuaa.edu.cn (X.Z.)

**Keywords:** enhanced spatial smoothing, Direction of Arrival (DOA) estimation, coherent signals, signal subspace

## Abstract

This paper investigates the direction of arrival (DOA) estimation of coherent signals with a moving coprime array (MCA). Spatial smoothing techniques are often used to deal with the covariance matrix of coherent signals, but they cannot be used in sparse arrays. Therefore, super-resolution algorithms such as multiple signal classification (MUSIC) cannot be applied in the DOA estimation of coherent signals in sparse arrays. In this study, we propose an enhanced spatial smoothing method specifically designed for MCA. Firstly, we combine the signals received by the MCA at different times, which can be regarded as a sparse array with a larger number of array sensors. Secondly, we describe how to compute the covariance matrix, derive the signal subspace by eigenvalue decomposition, and prove that the signal subspace is also equivalent to a received signal. Thirdly, we apply enhanced spatial smoothing to the signal subspace and construct a rank recovered covariance matrix. Finally, the DOA of coherent signals are well estimated by the MUSIC algorithm. The simulation results validate the improved performance of the proposed algorithm compared with traditional methods, particularly in scenarios with low signal-to-noise ratios.

## 1. Introduction

The issue of Direction of Arrival (DOA) estimation is extensively researched in various fields, such as radar, sonar, navigation, and astronomy [1,2,3,4,5,6], in which antenna arrays are utilized to capture incoming signals. Several DOA estimation algorithms have been proposed by scholars using uniform linear arrays (ULAs), including multiple signal classification (MUSIC) [4], estimation of signal parameters via rotational invariance techniques (ESPRIT) [5], propagator method (PM) [7], CAPON [8], and parallel factor (PARAFAC) technique [9]. However, these methods are developed under the assumption of signal incoherence. In practical scenarios, signals are often coherent due to factors such as multipath propagation. The coherence introduces the issue of rank deficiency in the covariance matrix of the signal, thereby rendering the aforementioned methods ineffective.

To address the rank deficiency issue in the covariance matrix of coherent signals, several methods have been proposed. A generalized MUSIC algorithm was proposed in [10], and a subspace adaptation method was introduced in [11]. Nevertheless, these methods involve multidimensional searching, resulting in high computational complexity that is not suitable for practical applications. A well-known spatial smoothing technique, initially proposed in [12], has been further improved in subsequent works such as [13,14,15] (referred to as MSSP). This method treats the entire array as a series of overlapping subarrays and combines their covariance matrices to recover the rank. The spatial smoothing-based techniques have found numerous applications and undergone further improvements [16,17].

In addition to spatial smoothing, there are other methods available for recovering the covariance matrix of coherent signals. For instance, Refs. [18,19] propose new matrix construction methods that are independent of the coherence of the sources. Furthermore, Ref. [18] introduces a method based on Toeplitz matrix construction, which sacrifices array aperture to recover the rank. On the other hand, Ref. [19] presents a non-Toeplitz matrix method that can handle both coherent and incoherent source scenarios. An improved spatial smoothing technique called improved spatial smoothing (ISS) has gained considerable attention as proposed in [20,21]. These methods utilize not only the covariance matrix of each subarray but also the cross-covariance matrix between subarrays, thereby enhancing the DOA estimation performance. Furthermore, an enhanced spatial smoothing technique was introduced in [22], which further exploits the information in the signal subspace and exhibits stronger resistance to noise interference.

However, the previous methods are primarily developed for uniform linear arrays (ULAs) and may not be directly applicable to sparse arrays. Unlike ULAs, sparse arrays, such as nested arrays (NA) [23] and coprime arrays (CA) [24], have larger aperture arrays, which allow for more design flexibility as the inter-element spacing is not limited to half-wavelength. These sparse arrays offer enhanced performance and broader possibilities for DOA estimation.

Although that sparse array can increase the number of sensors in virtual array and greatly improve the array aperture compared with ULA, people still hope that the accuracy of DOA estimation can be improved and achieve a greater degree of freedom. Recently, scholars have tried to solve the problem of DOA estimation by utilizing the moving array. The moving array can significantly increase the degrees of freedom (DOF) of the array, particularly when combined with sparse array. This combination not only provides higher DOFs but also improves performance compared to the original array configuration. References [25,26,27] propose a non-hole-filled co-array that utilizes an extended array aperture. As the array moves, the previous gaps or “holes” in the array are filled, resulting in a larger effective aperture for the uniform linear array. But it is important to note that the above method is applicable only to incoherent signals. To address the coherence issue in moving coprime arrays, Ref. [28] utilizes the cross-correlation information of each subarray for decoherence processing. However, this approach involves convex optimization algorithms, which can increase the computational complexity of the DOA estimation process.

This paper combines the advantages of the above methods and proposes an enhanced spatial smoothing technique with low complexity. The DOA estimation performance in low signal-to-noise ratio (SNR) scenarios has been greatly improved, and the low complexity is more conducive to practical applications. This technique effectively utilizes the signal subspace of MCA, enabling rank recovery and extending its applicability to various sparse arrays. Then, we can apply the MUSIC method to estimate the DOA of the coherent signals after the proposed technique. In particular, our main contributions can be summarized as follows:

(1) We propose an enhanced spatial smoothing technique applied to MCA, using the mobility of the array to form a sparse array with more array sensors, and the DOFs are greatly improved.
(2) The proposed technique exhibits significant improvement in noise interference resistance by leveraging the signal subspace to construct a new covariance matrix. The DOA estimation performance surpasses that of the SS-MUSIC algorithm in low SNR scenarios.
(3) We compare the estimated performance and runtime of the proposed algorithm with classical compressed sensing algorithms to demonstrate that the proposed algorithm outperforms the compressed sensing algorithms in terms of estimation performance. Additionally, the complexity of the proposed algorithm is significantly lower than that of the compressed sensing algorithms.

The rest of the paper is organized as follows: In Section 2, we provide the mathematical model of moving coprime array and characteristics of MCA. In Section 3, we present the detailed steps of the proposed algorithm. Section 4 analyzes the CRB of the array. Section 5 analysis the theoretical performance of the proposed algorithm by simulation. In Section 6, we conclude this paper.

Notations: Scalars, vectors, matrices, and sets are represented by lowercase letters a, lowercase letters in boldface a, uppercase letters in boldface A, and letters in blackboard boldface A, respectively. An R-dimensional vector n is denoted by ${\left[n^{\left(1\right)},n^{\left(2\right)},\cdots ,n^{\left(R\right)}\right]}^{T}$, where $n^{\left(r\right)}$ is the rth coordinate. $A^{T}$ is the transpose of A, and $A^{H}$ is the complex conjugate transpose of A. $diag\left(\cdot \right)$ denotes the matrix formed by the diagonal elements of the matrix. ${\left[A\right]}_{i,j}$ represents the $\left(i,j\right)$ entry of A. $A\left(a:b,c:d\right)$ refers to the matrix formed by selecting the elements of matrix A that are located in rows a to b and columns c to d.

## 2. Mathematical Model

**Figure 1:**
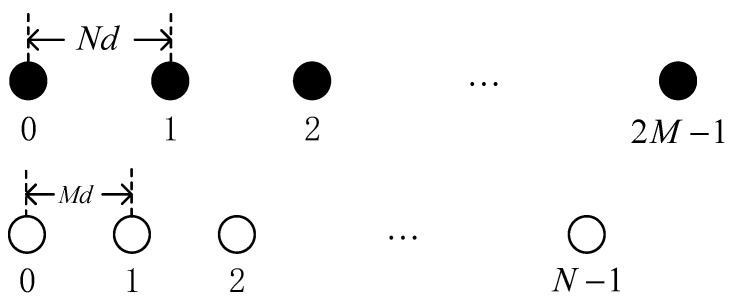
Coprime array.

Consider a coprime array, depicted in Figure 1, composed of two sparse ULAs with a total of 2M+N−1 sensors. Each ULA consists of 2M and N sensors, respectively, with inter-sensor spacings of Nd and Md (d being the half wavelength, M<N). The leftmost sensor of the two ULAs is denoted as the reference sensor, and the location of the coprime array sensor is [24]

(1) $$\begin{array}{c} S=\left\{2M\left(N-1\right)d-mNd|0\leq m\leq 2M-1\right\}\\ \cup \left\{2M\left(N-1\right)d-nMd|0\leq n\leq N-1\right\}. \end{array}$$

Define $l_{i},i=1,2,\cdots ,\left|S\right|$ as the location of ith sensor in coprime array with $l_{1}=0$ and $\left|S\right|=2M+N-1$.

Consider that the array is at rest at time instant $t_{1}$. Assume that the noise generated when receiving the signal is additive white Gaussian noise with zero mean. There are K far-field narrowband coherent signals impinged on the above array with different directions $\theta ={\left[\theta_{1},\theta_{2},\cdots ,\theta_{K}\right]}^{T}$. The signal we observed from the array may be expressed as [28]

(2) $$\begin{array}{c} x_{m0}\left(t\right)=\sum_{k=1}^{K}\alpha_{k}a\left(\theta_{k}\right)s\left(t\right)+n_{m0}\left(t\right)\\ =A\alpha s\left(t\right)+n_{m0}\left(t\right), \end{array}$$

where $A=\left[a\left(\theta_{1}\right),a\left(\theta_{2}\right),\cdots ,a\left(\theta_{K}\right)\right]$ represents the direction matrix. $a\left(\theta_{i}\right)={\left[1,e^{j2\pi l_{2}\sin\theta_{i}/\lambda },\cdots ,e^{j2\pi l_{\left|S\right|}\sin\theta_{i}/\lambda }\right]}^{T}$ is the direction vector of the ith signal. λ denotes the wavelength of the signal. $s\left(t\right)$ is the signal waveform. $\alpha ={\left[\alpha_{1},\alpha_{2},\cdots ,\alpha_{K}\right]}^{T}$ means the nonzero coherence coefficient vector. $n_{m0}\left(t\right)$ denotes the additive white Gaussian noise.

Assuming that at time $t_{1}$, the array receives the signal of J snapshots, the model of the received signal can be expressed as

(3) $$X_{m0}=A\alpha s+N_{m0},$$

where $s\in {\mathbb{C}}^{1\times J}$ is the signal waveform vector. $N_{m0}$ denotes the additive white Gaussian noise.

Suppose the above coprime array is moving along the array axis with velocity v. It is assumed that the DOA value of the far-field signals does not change within a short time Δt. In an isotropic underwater medium [29], the narrowband signal has a significantly smaller frequency band compared to the carrier value. As a result, the change in the signal is relatively slow, allowing us to disregard the variation in signal envelope across each array sensor. In other words, when the ideal conditions are assumed, we can assume that the amplitude of the reference signal does not change during the moving of the array, but its phase is rotated by $\phi_{\Delta t}$, which can be written as

(4) $$s\left(t_{2}\right)=s\left(t_{1}+\Delta t\right)=e^{j\phi_{\Delta t}}s\left(t_{1}\right).$$

**Figure 2:**
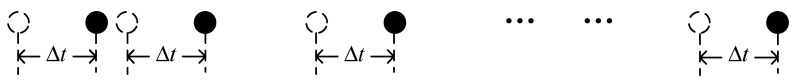
The position of the array at time $t_{2}$.

Figure 2 illustrates the position of the array relative to the initial time $t_{1}$ after time instant Δt, with the white hollow circle representing the original array position and the black solid circle indicating its position at time $t_{2}$.

Consequently, at time instant $t_{2}\left(t_{2}=t_{1}+\Delta t\right)$, the received data vector can be modeled as

(5) $$\begin{array}{cc} X_{m1} & =\sum_{k=1}^{K}\alpha_{k}e^{j2\pi v\Delta t\sin\theta_{k}/\lambda }a\left(\theta_{k}\right)e^{j\Delta \phi }s+N_{m1}\\ & =AD\Phi \alpha s+N_{m1}, \end{array}$$

where $D=diag\left(e^{j2\pi v\Delta t\sin\theta_{1}/\lambda },e^{j2\pi v\Delta t\sin\theta_{2}/\lambda },\cdots ,e^{j2\pi v\Delta t\sin\theta_{K}/\lambda }\right)$.$\Phi =diag\left(e^{j\phi_{\Delta t}},\cdots ,e^{j\phi_{\Delta t}}\right)$.$N_{m1}$ represents the additive white Gaussian noise.

## 3. Proposed Algorithm

In this section, we first present the application of MCA properties for the creation of an extended sparse array to receive signals. Subsequently, we examined the direct implementation of spatial smoothing techniques to handle the received signals. Finally, we put forth an improved spatial smoothing technique and substantiated its viability.

### 3.1. Spatial Smoothing Technique

According to the above mathematical model, it can be deduced that when the MCA moves for the Lth time, the received data can be modeled as

(6) $$X_{mL}=AD^{L}\Phi^{L}\alpha s+N_{mL},$$

where $N_{mL}$ represents the additive white Gaussian noise.

Combining all received signals, we get

(7) $$Y=\left[\begin{array}{c} X_{m0}\\ X_{m1}\\ \vdots \\ X_{mL} \end{array}\right]=\left[\begin{array}{c} A\alpha s+N_{m0}\\ AD\Phi \alpha s+N_{m1}\\ \vdots \\ AD^{L}\Phi^{L}\alpha s+N_{mL} \end{array}\right]=\left[\begin{array}{c} A\\ AD\Phi \\ \vdots \\ AD^{L}\Phi^{L} \end{array}\right]\alpha s+N_{y}=A_{y}\alpha s+N_{y},$$

where

(8) $$A_{y}=\left[\begin{array}{c} A\\ AD\Phi \\ \vdots \\ AD^{L}\Phi^{L} \end{array}\right].$$

$N_{y}$ represents additive white Gaussian noise with zero mean and variance $\sigma_{y}^{2}$.

The covariance matrix of Y can be given by [4]

(9) $$\begin{array}{cc} R_{y} & =E\left[YY^{H}\right]\\ & =A_{y}\alpha ss^{H}\alpha^{H}A_{y}^{H}+\sigma_{y}^{2}I_{L\left|S\right|}\\ & =A_{y}R_{S}A_{y}^{H}+\sigma_{y}^{2}I_{L\left|S\right|}, \end{array}$$

where $R_{S}=\alpha ss^{H}\alpha^{H}\in {\mathbb{C}}^{K\times K}$ represents the covariance matrix of the coherent signals. $I_{L\left|S\right|}\in {\mathbb{C}}^{L\left|S\right|\times L\left|S\right|}$ is the identity matrix.

Equation (8) is the theoretical formula for calculating the covariance matrix. In practice, we approximate the covariance matrix by finite snapshots, which can be written as

(10) $$R_{y}=YY_{}^{H}/J$$

The spatial smoothing technique is to divide the original array into multiple same sub-arrays, and the MCA can be regarded as an array composed of multiple identical CAs. The autocorrelation matrix of each measurement data $X_{mi}$ can be expressed as [12]

(11) $$\begin{array}{cc} R_{i} & =E\left[X_{mi}X_{mi}^{H}\right]\\ & =AD^{i}\Phi^{i}\alpha ss^{H}\alpha^{H}{\left(\Phi^{i}\right)}^{H}{\left(D^{i}\right)}^{H}A_{}^{H}+\sigma_{i}^{2}I_{\left|S\right|}\\ & =A{\left(D\Phi \right)}^{i}R_{S}{\left({\left(D\Phi \right)}^{i}\right)}^{H}A_{}^{H}+\sigma_{i}^{2}I_{\left|S\right|}. \end{array}$$

Summing and taking the average gives

(12) $$\begin{array}{cc} R_{y}^{f} & =\frac{1}{L}\sum_{i=1}^{L}R_{i}\\ & =\frac{1}{L}\sum_{i=1}^{L}A{\left(D\Phi \right)}^{i}R_{S}{\left({\left(D\Phi \right)}^{i}\right)}^{H}\\ & =A\left(\frac{1}{L}\sum_{i=1}^{L}{\left(D\Phi \right)}^{i}R_{S}{\left({\left(D\Phi \right)}^{i}\right)}^{H}\right)A_{}^{H}+\sigma_{i}^{2}I_{\left|S\right|}\\ & =AR_{SS}A_{}^{H}, \end{array}$$

where $R_{SS}=\frac{1}{L}\sum_{i=1}^{L}{\left(D\Phi \right)}^{i}R_{S}{\left({\left(D\Phi \right)}^{i}\right)}^{H}$.

We can find that

$$R_{i}=R_{y}\left(1+\left(i-1\right)\left|S\right|:\left|S\right|+\left(i-1\right)\left|S\right|,1+\left(i-1\right)\left|S\right|:\left|S\right|+\left(i-1\right)\left|S\right|\right).$$

Then, Equation (12) can be written as

(13) $$\begin{array}{cc} R_{y}^{f} & =\frac{1}{L}\sum_{i=1}^{L}R_{i}\\ & =\frac{1}{L}\sum_{i=1}^{L}R_{y}\left(1+\left(k-1\right)\left|S\right|:\left|S\right|+\left(k-1\right)\left|S\right|,1+\left(k-1\right)\left|S\right|:\left|S\right|+\left(k-1\right)\left|S\right|\right). \end{array}$$

Assume that the number of sources is known and L>K. Then, we can demonstrate the rank of $R_{SS}$ is equivalent to the number of signals according to [14]. As a result, the rank of Equation (12) is also equal to the number of signals, then we can utilize MUSIC to estimate the DOAs in Equation (13).

### 3.2. Enhanced Spatial Smoothing Technique

This section proposes an alternative method for utilizing the covariance matrix called enhanced spatial smoothing. Firstly, we perform eigen decomposition on the covariance matrix in Equation (8). Next, we construct a new covariance matrix by utilizing the eigenvector corresponding to the maximum eigenvalue. The advantage of this approach lies in fully exploiting the signal subspace, thereby offering better resistance to noise interference compared to the spatial smoothing technique.

Equation (10) can be decomposed using eigenvalues as follows [22]

(14) $$\begin{array}{cc} R_{y} & =E\left[YY^{H}\right]\\ & =\Gamma_{s}+\Gamma_{n}\\ & =\lambda_{s}u_{s}^{}u_{s}^{H}+U_{n}\Lambda_{n}U_{n}^{H}, \end{array}$$

where $\lambda_{s}$ is the largest eigenvalue and $u_{s}$ is its corresponding eigenvector. $\Lambda_{n}$ is a diagonal matrix composed of the remaining $\left|S\right|-1$ smaller eigenvalues, $U_{n}$ is the matrix of its corresponding eigenvectors. The expression of the matrix $\Gamma_{s}$ differs between coherent signals and non-fully correlated signals. This paper only discusses the case of fully coherent signals.

Since

(15) $$u_{s}^{}u_{s}^{H}+U_{n}U_{n}^{H}=I_{ML}.$$

We can get

(16) $$\begin{array}{cc} A_{y}R_{S}A_{y}^{H} & =R_{y}-\sigma_{y}^{2}I_{ML}\\ & =\lambda_{s}u_{s}^{}u_{s}^{H}+\sigma_{y}^{2}U_{n}U_{n}^{H}-\sigma_{y}^{2}I_{ML}\\ & =\left(\lambda_{s}-\sigma_{y}^{2}\right)u_{s}^{}u_{s}^{H}. \end{array}$$

Therefore, we can express $u_{s}$ as

(17) $$\begin{array}{cc} u_{s}^{} & =\frac{1}{\lambda_{s}-\sigma_{Y}^{2}}A_{y}SA_{y}^{H}u_{s}^{}\\ & =A_{y}^{}t, \end{array}$$

where $t=\frac{1}{\lambda_{s}-\sigma_{y}^{2}}SA_{y}^{H}u_{s}^{}$ is a $\left(K\times 1\right)$ vector.

From the derivation of the above equation, we can consider $u_{s}$ as a new received signal. Therefore, we take M consecutive elements of $u_{s}$, denoted as

(18) $$\begin{array}{cc} v_{i} & =u_{s}\left(i:i+\left|S\right|-1\right)\\ & =AD^{i-1}\Phi^{i-1}t,i=1,1+\left|S\right|,\cdots ,1+\left(L-1\right)\left|S\right|. \end{array}$$

In this case, $v_{i}$ can be considered equivalent to each sub-array in spatial smoothing, with the same array manifold as the original array. Define the cross-correlation operator as

(19) $$\begin{array}{cc} \Gamma_{ij} & =v_{i}v_{j}^{H}\\ & =u_{s}\left(i:i+\left|S\right|-1\right)u_{s}^{H}\left(j:j+\left|S\right|-1\right)\\ & =AD^{i-1}\Phi^{i-1}tt^{H}{\left(D^{j-1}\Phi^{j-1}\right)}^{H}A_{}^{H}. \end{array}$$

Equation (19) represents the covariance matrix of the signals with direction matrix A. Finally, we compute the rank-recovered covariance matrix using the following formula

(20) $$R=\frac{1}{L}\sum_{i=1}^{L}\sum_{j=1}^{L}\Gamma_{ij}\Gamma_{ji}.$$

Expanding Equation (20), we obtain the following expression

(21) $$\begin{array}{cc} R & =\frac{1}{L}\sum_{i=1}^{L}\sum_{j=1}^{L}\Gamma_{ij}\Gamma_{ji}\\ & =\frac{1}{L}\sum_{i=1}^{L}\sum_{j=1}^{L}\left(v_{i}v_{j}^{H}v_{j}v_{i}^{H}\right)\\ & =\frac{1}{L}\sum_{i=1}^{L}\sum_{j=1}^{L}\left(AD^{i-1}tt^{H}{\left(D^{j-1}\Phi^{j-1}\right)}^{H}A_{}^{H}AD^{j-1}tt^{H}{\left(D^{i-1}\Phi^{i-1}\right)}^{H}A_{}^{H}\right)\\ & =A\left[\frac{1}{L}\sum_{i=1}^{L}\sum_{j=1}^{L}\left(D^{i-1}tt^{H}{\left(D^{j-1}\Phi^{j-1}\right)}^{H}A_{}^{H}A_{}D^{j-1}tt^{H}{\left(D^{i-1}\Phi^{i-1}\right)}^{H}\right)\right]A_{}^{H}\\ & =AR_{ESS}A_{}^{H}, \end{array}$$

where $R_{ESS}=\frac{1}{L}\sum_{i=1}^{L}\sum_{j=1}^{L}\left(D^{i-1}tt^{H}{\left(D^{j-1}\Phi^{j-1}\right)}^{H}A_{}^{H}A_{}D^{j-1}tt^{H}{\left(D^{i-1}\Phi^{i-1}\right)}^{H}\right)$.

According to [12], the covariance matrix $R_{ESS}$ is of rank K, we can conclude that the rank of $AR_{ESS}A_{}^{H}$ is K. Therefore, the rank of R is equal to K, which means that the proposed method can restore the rank of the data covariance matrix.

The application of this method is suitable for various sparse arrays. It is crucial to highlight that the array needs to meet the requirement of having an inter-sensor spacing of half-wavelength or having array elements with relatively prime positions [30]. Only under these circumstances can we obtain an unambiguous estimation of the final DOA. In comparison to conventional methods, the proposed algorithm maximizes the utilization of the signal subspace and is less affected by noise. Then, we can estimate the DOA from the given R more effectively.

The main steps of the proposed algorithm are summarized as follows:

Step1: Combine the received signals into the matrix Y according to Equation (7);

Step2: Compute the covariance matrix of Y and obtain the eigenvector corresponding to its largest eigenvalue $\lambda_{s}$;

Step3: Calculate different $v_{i}$ according to Equation (18);

Step4: Calculate the covariance matrix R according to Equations (19) and (20);

Step5: Estimate the DOAs by the MUSIC algorithm.

## 4. Performance Analysis

### 4.1. Complexity Analysis

In this section, a number of well-known compressive sensing algorithms were chosen for analysis. These algorithms include sparse Bayesian learning (SBL) [31], nuclear norm minimization (NNM) [32], as well as classical spatial smoothing algorithms such as SS-MUSIC and SS-CAPON [8]. A thorough evaluation was conducted, comparing the performance of these algorithms with our own proposed algorithm.

The complexity of the algorithm in this article can be divided into several parts. Firstly, the number of multiplications required to compute the covariance matrix of the signal is ${\left|S\right|}^{2}L^{2}J$. The number of multiplications for performing eigenvalue decomposition on the covariance matrix is ${\left|S\right|}^{3}L^{3}$. The number of multiplications for constructing the new covariance matrix is $\left|S\right|L^{2}$. Finally, the number of multiplications for solving the direction of arrival (DOA) using the MUSIC algorithm are as follows: ${\left|S\right|}^{2}J$ for constructing the covariance matrix, ${\left|S\right|}^{3}$ for eigenvalue decomposition, ${\left|S\right|}^{2}\left(K+2\right)G$ for peak searching in the spectrum, and G represents the number of grid points. Therefore, the total number of multiplications for the proposed algorithm is

(22) $${\left|S\right|}^{2}J\left(L^{2}+1\right)+{\left|S\right|}^{3}\left(L^{3}+1\right)+\left|S\right|L^{2}+{\left|S\right|}^{2}\left(K+2\right)G.$$

The complexity comparison of SS-MUSIC algorithm, the SS-CAPON algorithm, and the proposed algorithm can be shown in Table 1.

The number of multiplications for compressed sensing algorithms like NNM cannot be accurately represented due to the convex optimization process involved, as well as the iteration count of SBL being dependent on the convergence threshold. To compare the runtime of these algorithms, we execute them one, ten, fifty, and five hundred times, as indicated in Table 2. According to the table, the SS-MUSIC algorithm has the shortest runtime. Our proposed algorithm’s runtime is very similar to that of the SS-MUSIC algorithm, with negligible differences. In comparison to compressive sensing algorithms, our method has lower computational complexity and requires fewer array sensors, making it more suitable for practical applications with limited resources.

### 4.2. Crarmer-Rao Bound (CRB)

The Cramer-Rao Bound (CRB) matrix can be modeled as [33]

(23) CRB=σn22JReDHΠA⊥DP^T−1,

where A means the manifold matrix of array, $\Pi_{A}^{⊥}=I-A{\left(A^{H}A\right)}^{-1}A^{H}$ is the orthogonal projection of A, $\hat{P}=1/J\sum_{t=1}^{J}s\left(t\right)s^{H}\left(t\right)$, $\sigma_{n}^{2}$ represents the average power of signal source, and D can be modeled as

(24) $$D=\left[\frac{\partial a\left(\theta_{1}\right)}{\partial \theta_{1}},\frac{\partial a\left(\theta_{2}\right)}{\partial \theta_{2}},\cdots ,\frac{\partial a\left(\theta_{K}\right)}{\partial \theta_{K}}\right],$$

where $a\left(\theta_{K}\right)$ is steering vector.

### 4.3. The Advantages of the Proposed Technique

The proposed technique exhibits significantly reduced complexity compared to the compressed sensing algorithm. Additionally, by conducting space smoothing in the signal subspace, the correlation between DOA estimation and the noise subspace was minimized, resulting in superior anti-noise interference capabilities compared to compressed sensing algorithms. In other words, even in scenarios with low SNRs, the DOA of coherent signal can still be accurately estimated using this technique.

## 5. Simulation Results

Consider a coprime array with parameters set as M=4 and N=5, which moves along the array axis with a velocity of v=3.1 m/s. The received signals are collected at times 0 s, 0.25 s, 0.5 s, and 0.75 s. Two far-field narrowband coherent signals impinge on the array from 9.55° and 35.20°. Define the Root Mean Square Error (RMSE) of the DOA estimates as

(25) $$RMSE=\sqrt{\frac{1}{K}\frac{1}{G}\sum_{k=1}^{K}\sum_{g=1}^{G}{\left({\hat{\theta }}_{g,k}-\theta_{k}\right)}^{2}},$$

where G is the number of Monte Carlo trials. K denotes the total number of coherent sources. ${\hat{\theta }}_{j,k}$ means the gth estimate of true incidence $\theta_{k}$. In the following simulations, we set the number of Monte Carlo simulations as G=1000.

**Figure 3:**
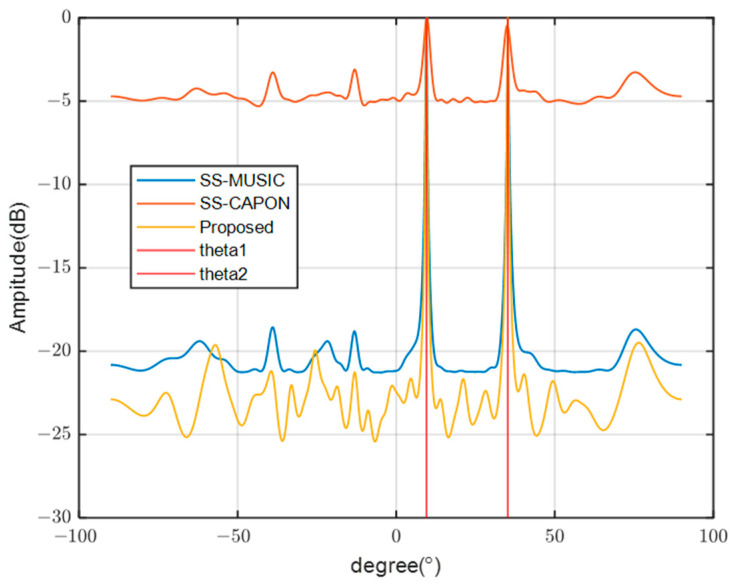
Comparison of spatial spectra between the proposed algorithm and other algorithms.

In order to evaluate peak searching capabilities, the proposed algorithm is compared to SS-MUSIC and SS-CAPON in Figure 3. The conditions for this comparison include a SNR of −5 dB and a total of 200 snapshots. Among these algorithms, SS-CAPON displays a peak that is 15 dB higher than the other two. On the other hand, the proposed algorithm showcases the most distinct peak with the least amount of sidelobes. This suggests that the proposed algorithm surpasses the other two in terms of its effectiveness in suppressing noise.

**Figure 4:**
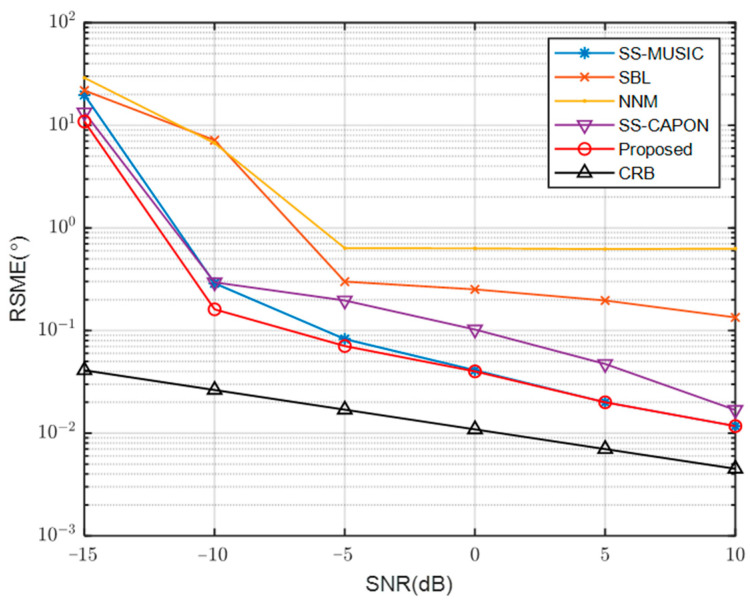
Comparison of RMSE of different algorithms with SNR.

Figure 4 presents a comparison of the RMSE curves for the mentioned algorithms, with varying SNR. The simulations were conducted with 200 snapshots. The proposed algorithm effectively utilizes the signal subspace and exhibits a significantly better resistance against noise interference compared to the other algorithms. Consequently, when the SNR is below zero, the RMSE of the proposed algorithm is the smallest among all algorithms. For SNR values above 0, the NNM algorithm’s curve shows a nearly linear trend, implying that its performance is not significantly affected by SNR improvement. Conversely, the RMSE curves of the other algorithms decrease as the SNR increases, indicating that higher SNR enhances their performance. Notably, the RMSE curve of the proposed algorithm aligns with the SS-MUSIC curve and demonstrates the lowest value among all algorithms, underscoring its superior performance under such circumstances.

**Figure 5:**
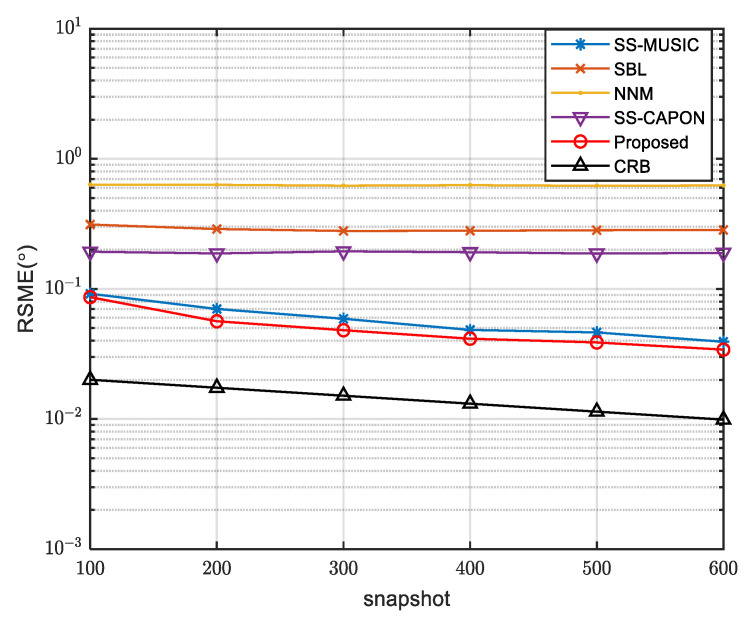
RMSE of different algorithms versus different snapshots.

In Figure 5, we conducted an analysis on the performance of various algorithms at different snapshot numbers. In the simulation where the SNR was fixed at −5 dB, we observed that the RMSE curves of NNM, SBL, and SS-CAPON did not decrease as the number of snapshots increased from 100 to 600. This implies that increasing the number of snapshots does not lead to an improvement in their performance. Conversely, the RMSE curves of the other algorithms demonstrated a decrease with an increasing number of snapshots, indicating an enhancement in their performance. Notably, our proposed algorithm achieved the lowest RMSE value among all the algorithms, highlighting its superior performance.

**Figure 6:**
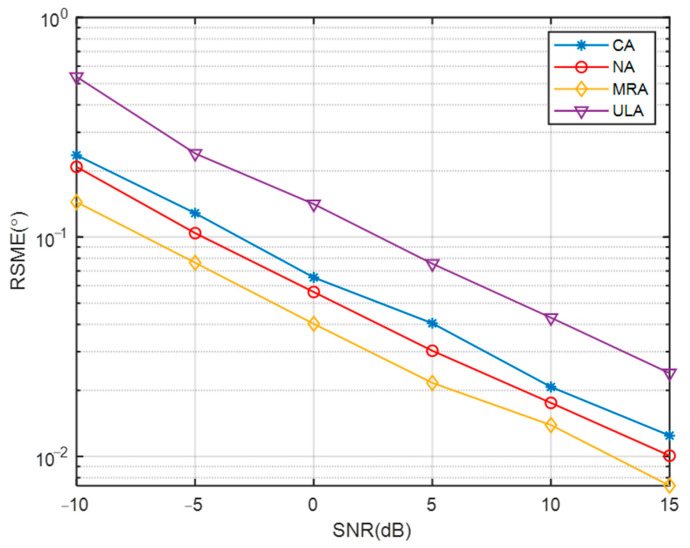
Comparison of RMSE of different arrays with SNR.

**Figure 7:**
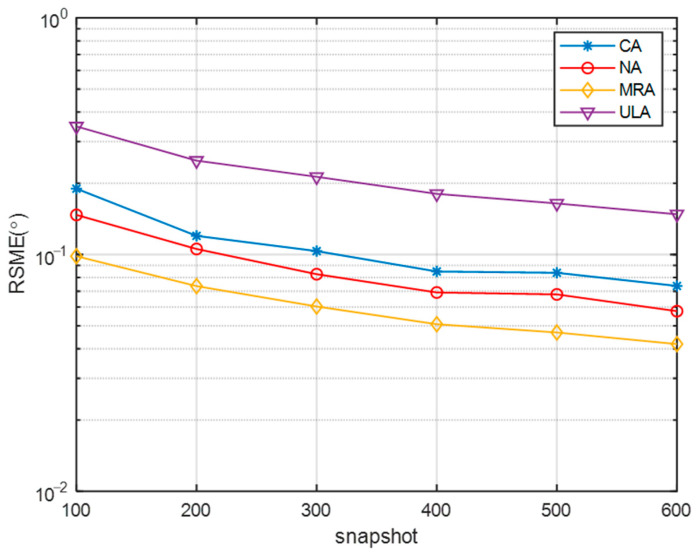
Comparison of RMSE of different arrays with snapshot.

Figure 6 and Figure 7 present an analysis of the proposed algorithm’s performance across various sparse arrays, considering changes in SNR and the number of snapshots. The number of sensors in the NA, MRA, ULA, and CA were set to be the same. In Figure 6, as the SNR increases, the RMSE curves of all algorithms decrease, with the DOA estimation performance ranking from highest to lowest as MRA, NA, CA, and ULA. This is attributed to the varying array apertures among the different arrays, with MRA having the largest aperture and yielding the best performance, while ULA had the smallest aperture and performed the poorest. In Figure 7, with a SNR of −5 dB, increasing the number of snapshots resulted in decreased RMSE values for all arrays, indicating improved DOA estimation performance. Consistent with the previous analysis, MRA, with the largest aperture, exhibits the best performance, while ULA, with the smallest aperture, has the highest RMSE.

**Figure 8:**
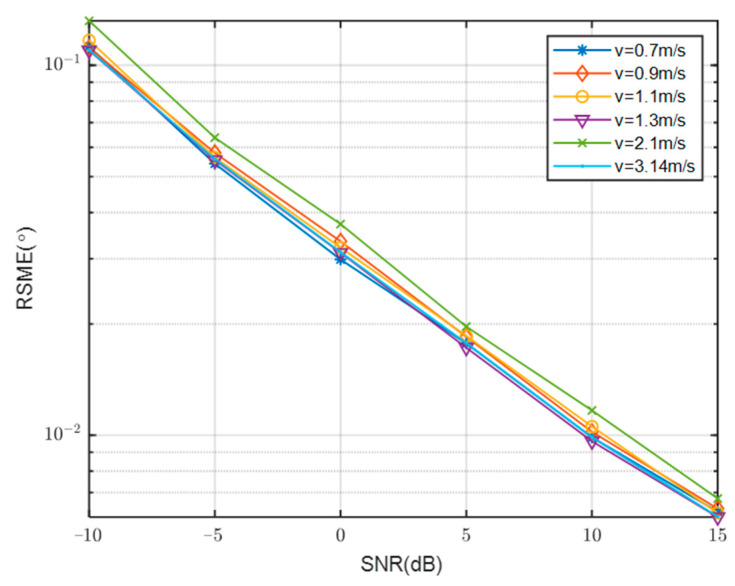
Comparison of RMSE of different speed with SNR.

Figure 8 presents a comparison of the RMSE curves with SNR at various velocities. Specifically, the velocities of 0.7 m/s, 0.9 m/s, 1.1 m/s, 1.3 m/s, 2.1 m/s, and 3.14 m/s were selected for analysis, while keeping other simulation conditions constant. The analysis reveals a clear influence of speed on the accuracy of DOA estimation. Ultimately, a velocity of 3.14 m/s was chosen as it corresponded to a relatively favorable curve.

**Figure 9:**
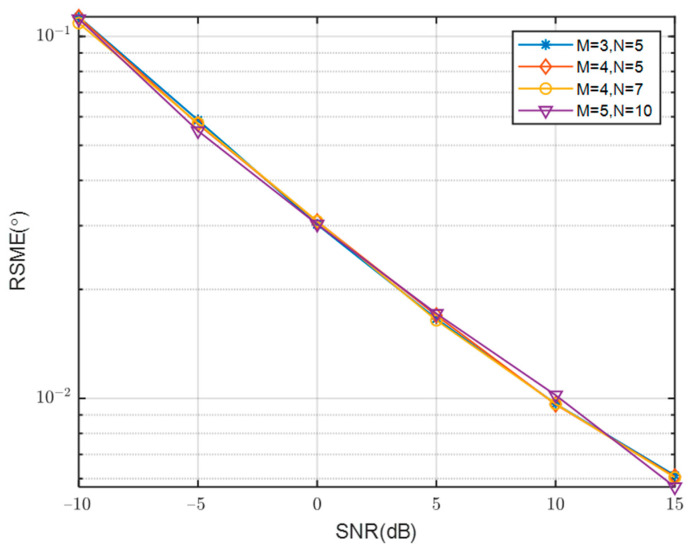
Comparison of RMSE of different array sensors with SNR.

**Figure 10:**
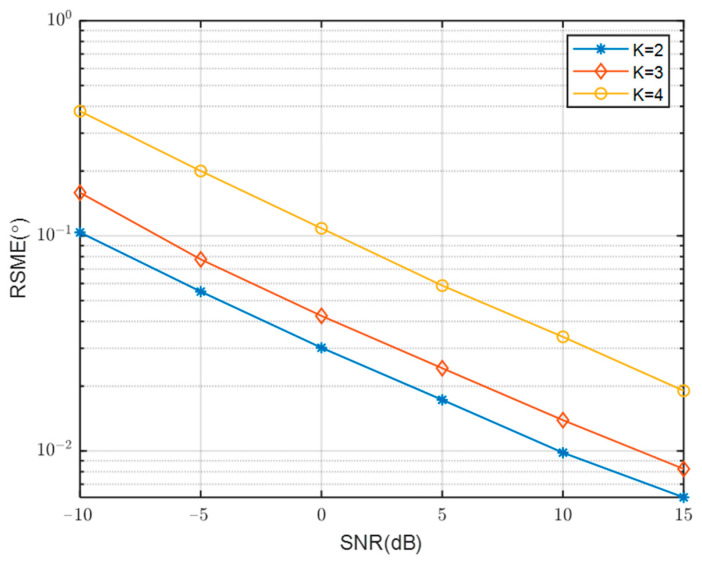
RMSE Comparison of different the number of emission sources with SNR.

The comparison of RMSE curves with SNR for different numbers of array sensors is presented in Figure 9. The simulation results indicate a close alignment between the RMSE curves and the number of array sensors. This can be attributed to the convergence of performance after spatial smoothing. The consistent outcomes support the selection of M = 4 and N = 5 as the final results. These values correspond to a relatively small number of array elements, allowing for the estimation of a larger number of sources simultaneously. Figure 10 presents a comparison of the RMSE change curves with SNR across varying numbers of signal sources. It is observed that the RMSE value increases as the number of signal sources continues to rise, indicating a degradation in the performance of DOA estimation with an increasing number of signal sources.

## 6. Conclusions

This paper introduces an enhanced spatial smoothing technique that is specifically tailored for sparse arrays. By taking advantage of the unique properties of the MCA, the technique optimizes the use of the signal subspace to effectively reduce interference. In comparison to traditional compressed sensing algorithms and spatial smoothing techniques, the suggested algorithm exhibits exceptional resistance to noise interference. It consistently delivers precise estimation of the DOA for incoming signals, even in situations with low SNR. In fact, we only focus on 1D-DOA cases. If it can be extended to an L-shaped array according to [34], then we can realize DOA estimation of coherent signal in 2D-DOA.

## Figures and Tables

**Table 1:** Comparison of complexity of different algorithms.

Algorithm	Computational Complexity
SS-MUSIC	${\left|S\right|}^{2}J\left(L+1\right)+{\left|S\right|}^{3}+{\left|S\right|}^{2}\left(K+2\right)G$
SS-CAPON	${\left|S\right|}^{2}J\left(L+1\right)+{\left|S\right|}^{3}G+2{\left|S\right|}^{2}G$
Proposed	${\left|S\right|}^{2}J\left(L^{2}+1\right)+{\left|S\right|}^{3}\left(L^{3}+1\right)+\left|S\right|L^{2}+{\left|S\right|}^{2}\left(K+2\right)G$

**Table 2:** Comparison of running times of different algorithms.

Monte Carlo	NNM	SBL	SS-CAPON	SS-MUSIC	Proposed
1	2.7168 s	0.2727 s	0.1325 s	0.0213 s	0.0221 s
10	17.4291 s	2.2816 s	1.2913 s	0.2158 s	0.2207 s
50	136.7953 s	12.8730 s	6.3633 s	1.0332 s	1.0667 s
500	1179.6828 s	149.7322 s	64.011 s	10.3451 s	10.6497 s

## Data Availability

Not applicable.
